# Supplementary material for: IKK2 controls the inflammatory potential of tissue-resident regulatory T cells in a murine gain of function model
Source: Nat Commun. 2024 Mar 25;15:2345. doi: 10.1038/s41467-024-45870-3 (PMC10963799; doi:10.1038/s41467-024-45870-3)
Supplement: Supplementary file 1 — Supplementary Information [file 41467_2024_45870_MOESM1_ESM.pdf]

SUPPLEMENTARY MATERIAL

IKK2 controls the inflammatory potential of tissue-resident regulatory T cells

Chelisa Cardinez[1,2,3,4], Yuwei Hao[1,2,3,5], Kristy Kwong[1,2,3,5], Ainsley R. Davies[1,2,3], Morgan Downes[1,3], Nadia A. Roberts[3], Jason D. Price[4], Raquel A. Hernandez[1,2,3], Jessica Lovell[3], Rochna Chand[1,2,3], Zhi-Ping Feng[6], Anselm Enders[1,3], Carola G. Vinuesa[1,7], Bahar Miraghazadeh[1,2,3], Matthew C. Cook[1,2,3,5]

[1]Centre for Personalized Immunology, John Curtin School of Medical Research, Australian National University, Canberra, ACT, Australia

[2]Translational Research Unit, The Canberra Hospital, Canberra, ACT, Australia

[3]Division of Immunology and Infectious Diseases, John Curtin School of Medical Research, Australian National University, Canberra, ACT, Australia

[4]Division of Genome Sciences and Cancer, John Curtin School of Medical Research, Australian National University, Canberra, ACT, Australia

[5] Cambridge Institute of Therapeutic Immunology and Infectious Disease, Department of Medicine, University of Cambridge, United Kingdom

[6] ANU Bioinformatics Consultancy, John Curtin School of Medical Research, Australian National University, Canberra, ACT, Australia

[7] Francis Crick Institute, London, United Kingdom

SUPPLEMENTARY FIGURES

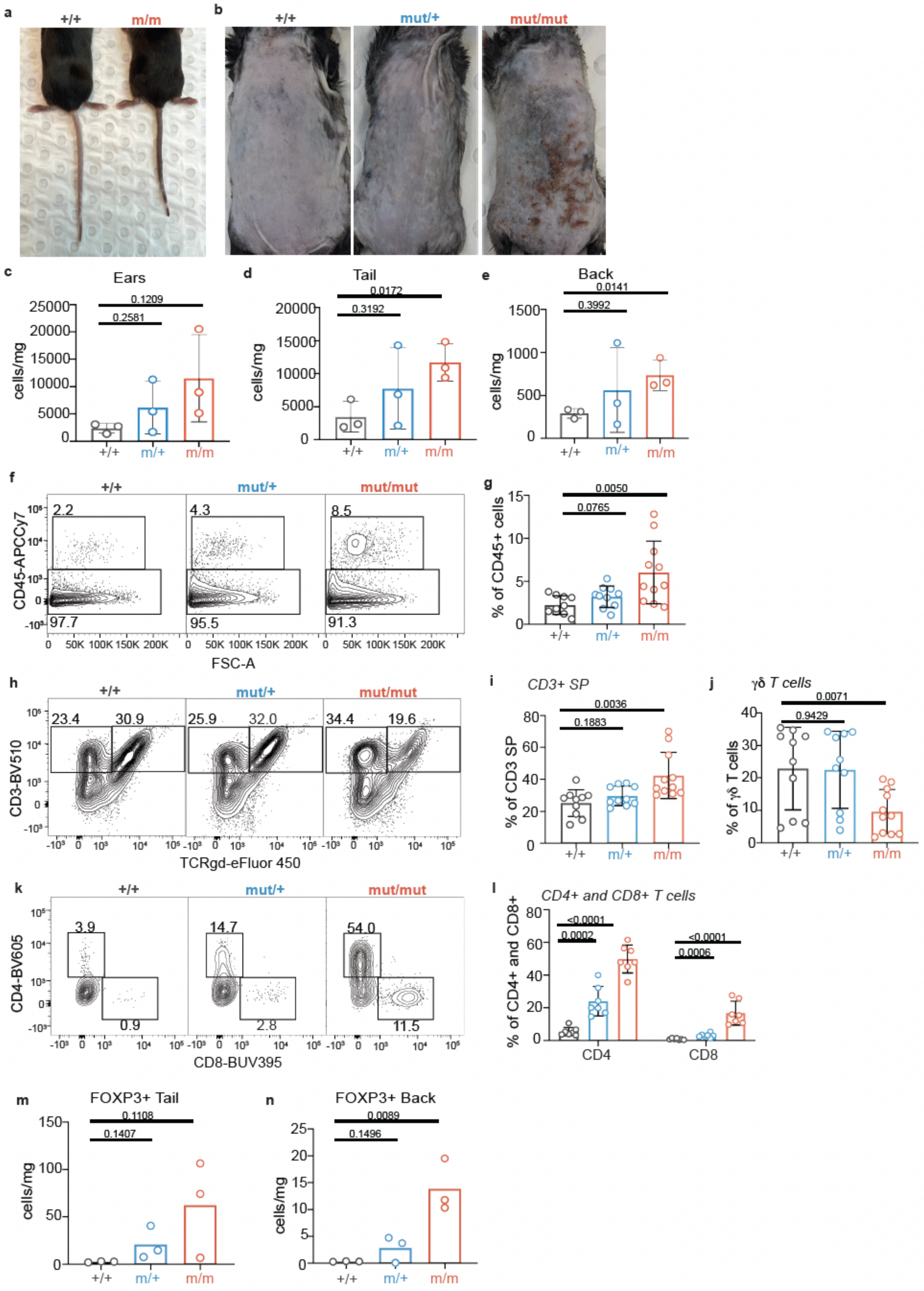

Supplementary figure 1: Cellular investigation of skin inflammation sites.

a-b. Representative images of tails (**a**) from *Ikbkb*$^{+/+}$ and *Ikbkb*$^{mut/mut}$ mice and back skin (**b**) from *Ikbkb*$^{+/+}$, *Ikbkb*$^{mut/+}$ and *Ikbkb*$^{mut/mut}$ mice, n=3/genotype. **c-e**. Total leukocyte counts recovered from digested ears (**c**), tails (**d**) and back (**e**) skin from mice of different *Ikbkb* genotypes, expressed as cells/milligram (mg). n=3/genotype. **f**. Representative flow cytometric analysis of CD45 and FSC-A live cells of each genotype, gated on CD45+ cells recovered from tail skin. **g**. Summary of CD45+ cells as a proportion of live cells. **h-l**. Analysis of T cells recovered from tail, showing representative flow cytometric analysis of CD3 and TCRγδ for each genotype (**h**), summaries of CD3+ γδTCR- T cells (**i**) and CD3+ TCRγδ+ T cells (**j**) as a proportion of CD45+ cells (n=10-11/genotype), and relative abundance of CD4 and CD8 T cells after gating on CD3+ (**k-l**) (n=7/genotype). m-n. Foxp3+ Treg cell counts in tail (m) and back skin (n) expressed as cells/mg of tissue for each genotype (n=3/genotype). In all plots, each symbol represents a biological replicate. P values were determined by one-way ANOVA with Bonferroni's multiple comparison test. Summary statistics indicate means +/- s.d.

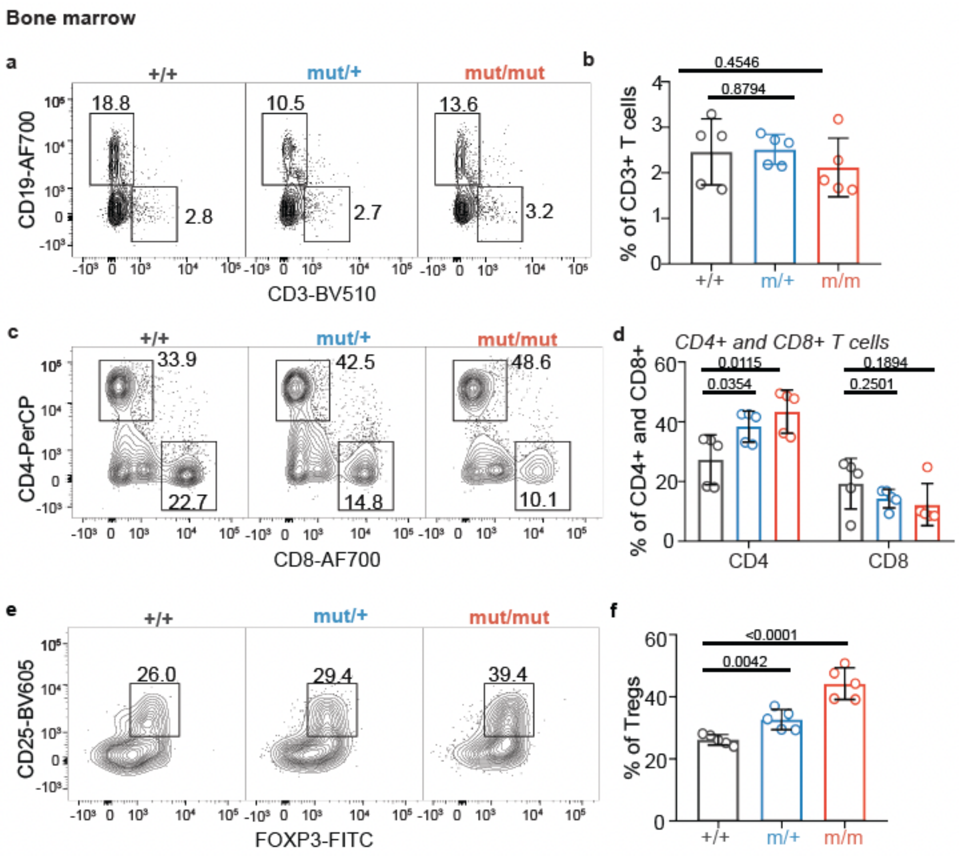

Supplementary figure 2: Immunophenotyping of bone marrow.

a. Representative flow cytometric analysis of CD19 and CD3 for each genotype, gated on live cells harvested from bone marrow (representative of 3 experiments). **b**. Summary of CD3+ T cells as a proportion of live cells. n=5/genotype. **c-d**. Flow cytometric analysis of CD4 and CD8 for each genotype, harvested from bone marrow and gated on CD3+ cells showing representative plots (**c**) and summary of results (**d**). n=4/genotype. **e-f**. Representative flow cytometric analysis of CD25+ Foxp3+ T cells gated on CD4+ T cells for each genotype, showing representative plots (**e**) and summary of CD25+ Foxp3+ Tregs as a proportion of CD4+ cells (**f**). n=5/genotype. In all plots, each symbol represents a biological replicate. *p* values were determined by one-way ANOVA with Bonferroni's multiple comparison test. Summary statistics indicate means +/- s.d.

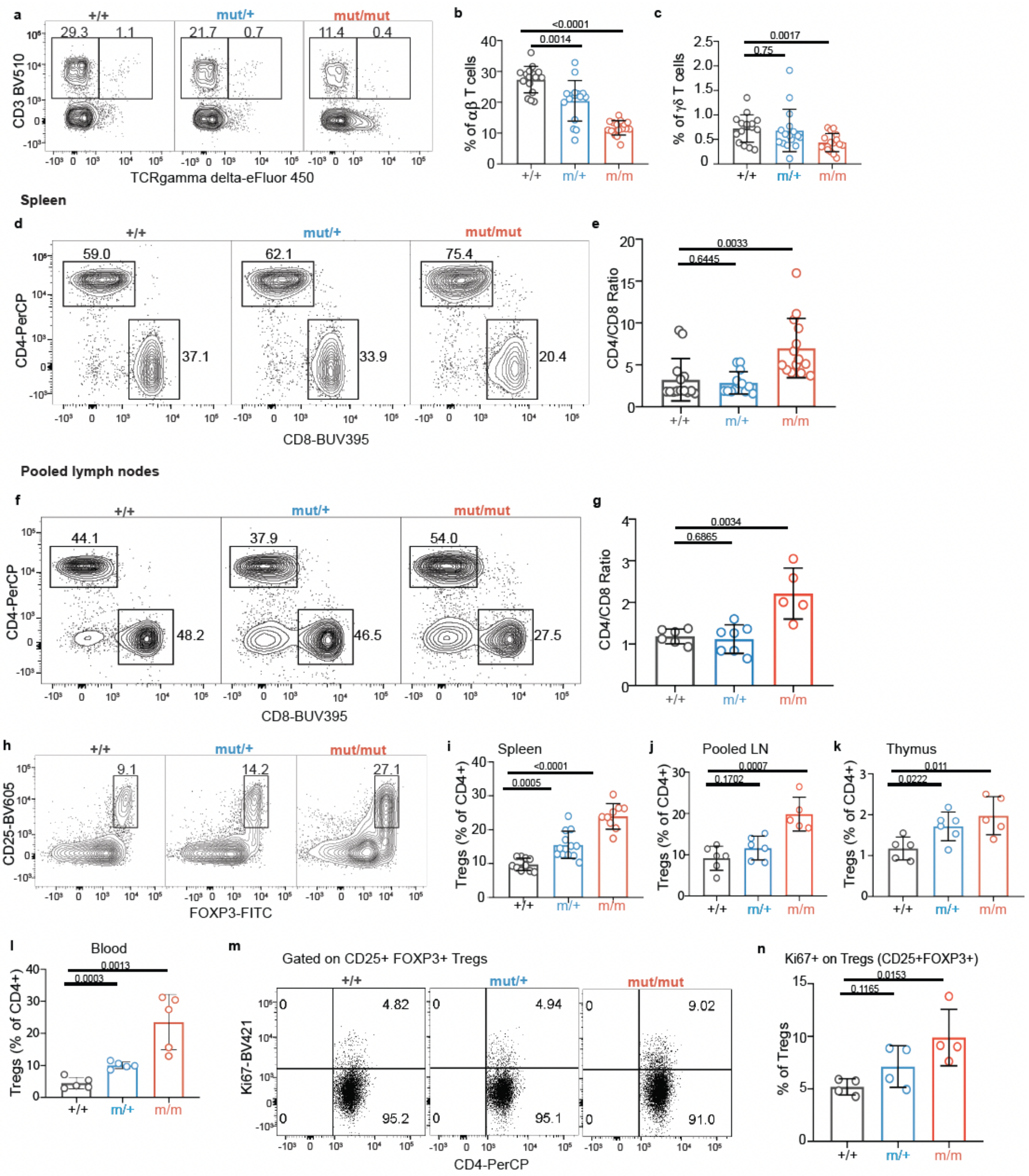

Supplementary figure 3: T cell analysis in spleen, lymph nodes, and thymus.

a-d. Analysis of splenic T cell composition showing representative flow cytometric plots for CD3 and TCRγδ for each *Ikbkb* genotype after gating on live cells (**a**), summaries of the proportion of CD3+ γδTCR- T cells (**b**) and CD3+ γδTCR+ cells. Representative flow cytometric analysis (**d**) and summary (**e**) of relative proportions of CD4 and CD8 expression by CD3+ T cells. n=13-16/genotype. **f-g.** Representative flow cytometric analysis (**f**) and summary (**g**) of relative proportions of CD4 and CD8 expression by CD3+

T cells in pooled lymph nodes. n=5-6/genotype. **h-l**. Analysis of relative abundance of Tregs showing representative flow cytometric analysis of CD25 and FOXP3 expression on CD4+ T cells from spleen (h), and summary results for spleen (n=9-11/genotype)(**i**), lymph nodes (n=5-6/genotype)(**j**), thymus (n=5-6/genotype) (**k**) and blood (n=5/genotype) (**l**) of mice of each *Ikbkb* genotype based on gating strategy shown in (**h**). **m-n**. Analysis of proliferating Tregs by Ki67 expression, showing representative plots (**m**) and summary (**n**) of Ki67+ Tregs as a proportion of CD25+Foxp3+ Tregs. In all plots, each symbol represents a biological replicate.

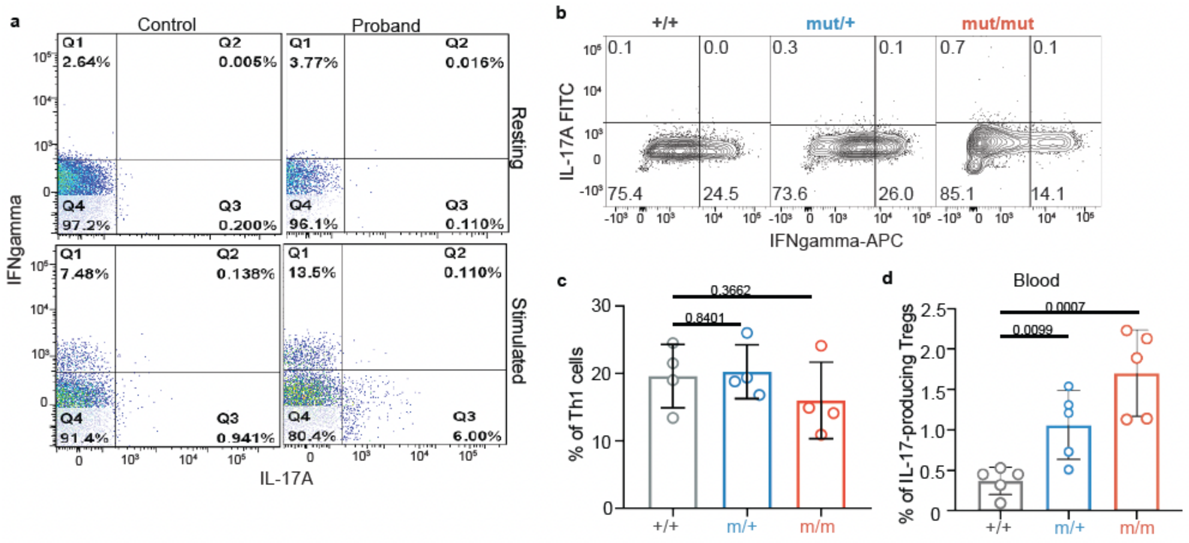

Supplementary figure 4: ***Ex vivo*** **cytokine analysis in patient and mice.**

a. Representative flow cytometric analysis of IFNγ and IL-17A expressing CD4+ T cells from healthy control and *IKBKB*^{V203I} patient in the presence or absence of stimulation (with PMA/Ionomycin). Representative of two independent experiments (technical replicates). **b-c.** Representative flow cytometric analysis (**b**) and summary (**c**) of IFNγ and IL-17A expressing CD4+ T cells after culturing native CD4+ T cells from mice of each *Ikbkb* genotype under Th1 differentiation conditions. n=4 donors/genotype. **d.** Summary of IL-17-producing Treg cells in the blood as a proportion of total Tregs (CD25+ Foxp3+) from mice of each *Ikbkb* genotype. n=5/genotype. In all plots, each symbol represents a biological replicate. *p* values were determined by one-way ANOVA with Bonferroni's multiple comparison test. Summary statistics indicate means +/- s.d.

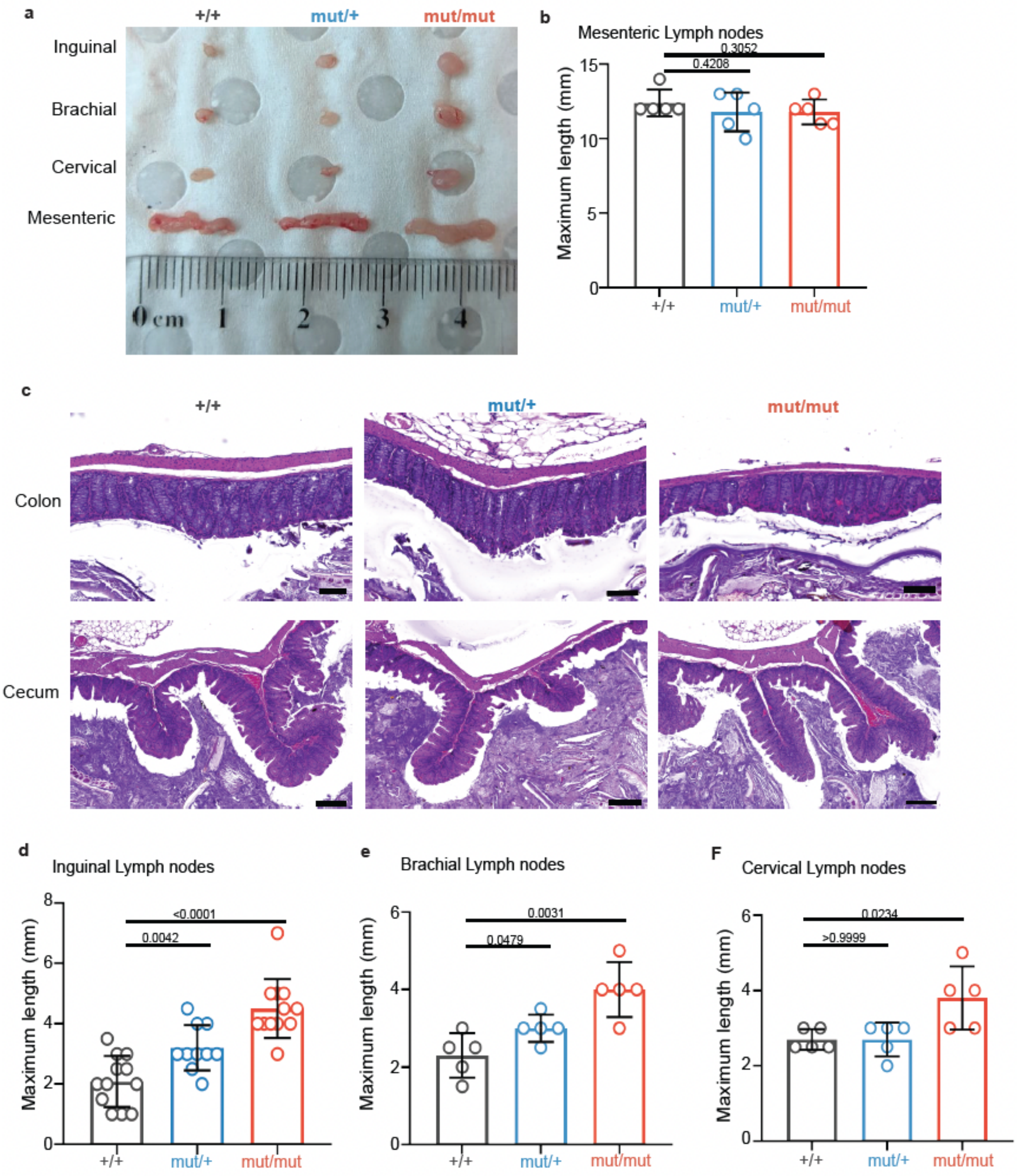

Supplementary figure 5: Lymph node size measurements for each genotype.

a-b. Analysis of lymphadenopathy showing representative images of lymph nodes from indicated region for mice of each *Ikbkb* genotype, with pooled results (**b**) for maximal lengths of mesenteric lymph nodes. n=5/genotype **c.** Representative sections of colon and cecum stained by H&E. Scale bars, 100µm for colon; 200µm for cecum. n= 3-4 mice per genotype. **d-f**. Summary of maximal lengths of inguinal (n=10/genotype) (**d**), brachial (n=5/genotype) (**e**), and cervical (n=5/genotype) (**f**) lymph nodes. In all plots, each

symbol represents a biological replicate. *p* values were determined by one-way ANOVA with Bonferroni's multiple comparison test. Summary statistics indicate means +/- s.d.

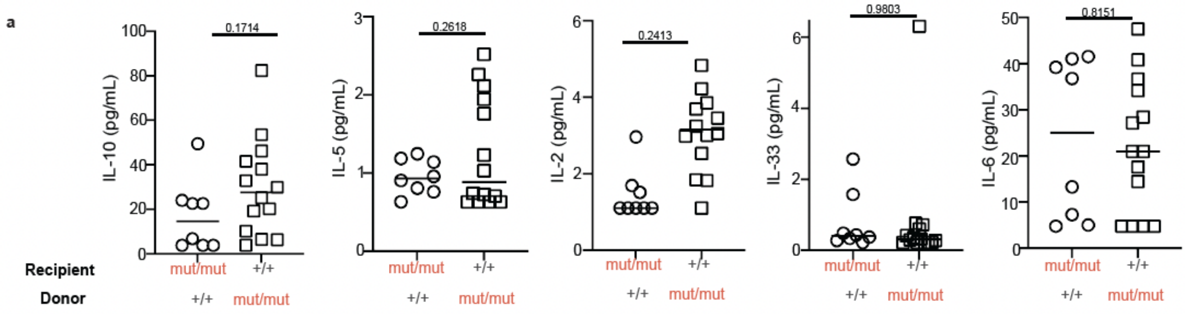

Supplementary figure 6: Serum cytokine analysis from reciprocal chimera mice. Serum cytokines from reciprocal chimera mice. In all plots, each symbol represents a biological replicate. *p* values were determined by Student’s t-test.

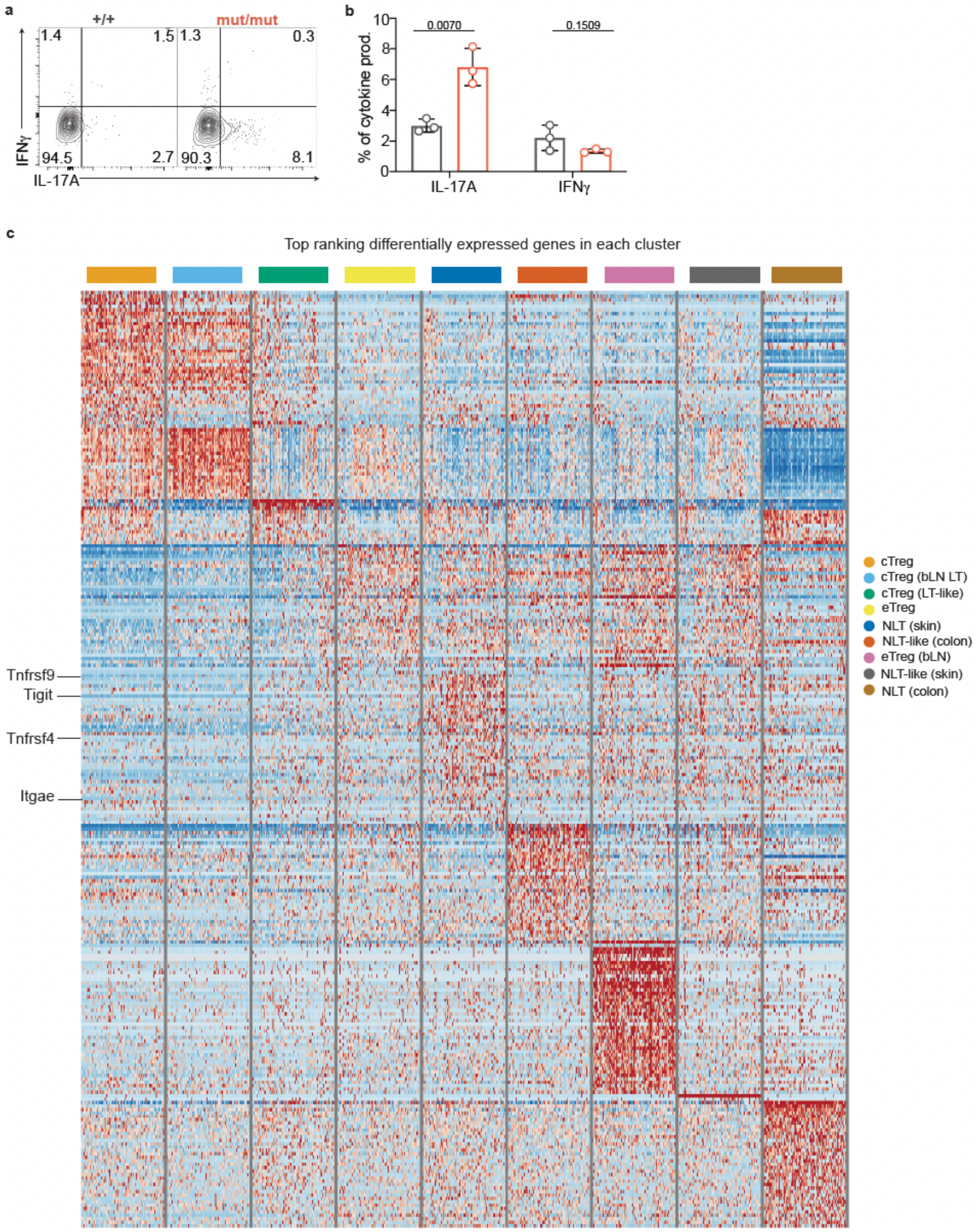

Supplementary figure 7: Top ranked differentially expressed genes in each Treg cluster.

a. Representative flow cytometric analysis for IL-17A and IFNγ of sorted WT and *Ikbkb*^{mut} Tregs from spleen (n=3). **b.** Summary of proportion of IL-17A+ and IFNγ Treg cells for

Ikbkb$^{mut/mut}$ or WT Tregs submitted for single-cell RNA-sequencing. Three mice for each genotype, with each data point representing a single mouse. Bar graph shows mean value with SD. Statistical analysis by two-tailed unpaired t-test. **c.** Heatmap (colour-scaled) showing top ranked differentially expressed genes in each identified Treg cluster. If more than 50 genes ranked in the cluster then top 50 were displayed. If less than 50 genes ranked in the cluster then all genes are displayed. Key genes identified in NLT (skin) Treg cluster include *Tnfrsf9, Tigit, Tnfrsf4,* and *Itgae.*

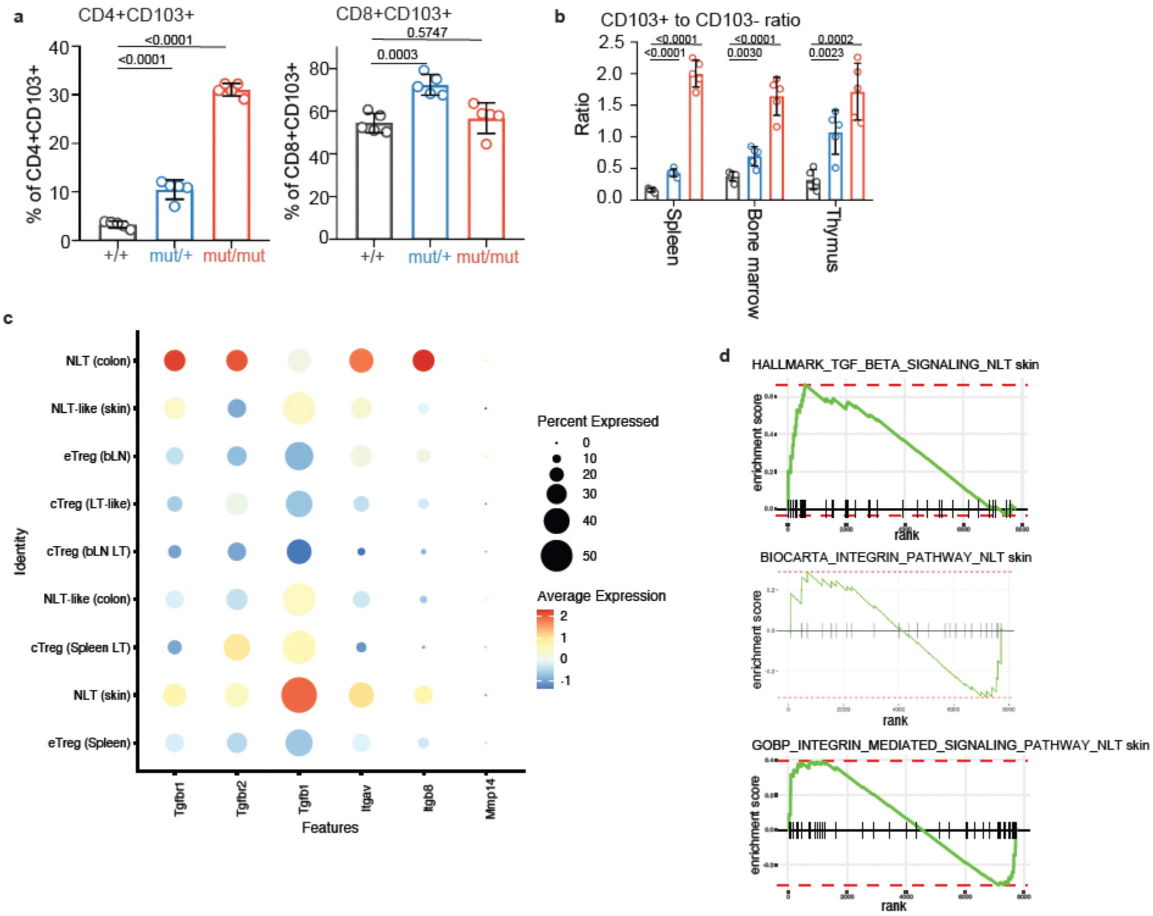

Supplementary figure 8: CD103 analysis and gene set enrichment analysis of NLT skin cluster.

a. Summary of $CD103^+$ cells as a proportion of $CD4^+$ and $CD8^+$ cells in spleen. n=5/genotype. **b**. Summary of $CD103^+$: $CD103^-$ ratio from Treg gate, obtained from spleen, bone marrow and thymus, analysed by Ikbkb genotype (*Ikbkb*$^{+/+}$, grey; *Ikbkb*$^{mut/+}$, blue; *Ikbkb*$^{mut/mut}$, red). n=3/group. **c.** scRNA-seq dot plot depicting the expression profiles of selected TGF-β pathway genes in each Treg cluster. **d.** Gene set enrichment analysis plots for the indicated TGF-β or integrin gene sets in the NLT skin cluster. HALLMARK_TGF_BETA_SIGNALING_NLT skin NES=1.6922, padj=0.0133. BIOCARTA_INTEGRIN_PATHWAY_NLT skin NES=-0.3308, padj=0.7910. GOBP_INTEGRIN_MEDIATED_SIGNALING_PATHWAY_NLT skin NES=0.3942, padj= 0.6950.
