# Supplementary material for: IKK2 controls the inflammatory potential of tissue-resident regulatory T cells in a murine gain of function model
Source: Nat Commun. 2024 Mar 25;15:2345. doi: 10.1038/s41467-024-45870-3 (PMC10963799; doi:10.1038/s41467-024-45870-3)
Supplement: Supplementary file 3 — Description of Additional Supplementary Files [file 41467_2024_45870_MOESM3_ESM.pdf]

Description of Additional Supplementary Files

Supplementary Data 1. Differentially expressed genes from bulk RNAseq comparing *Ikbkb*$^{mut/mut}$ and *Ikbkb*$^{WT/WT}$ ears. **a**. Summary statistics. Gene expression was normalized using trimmed mean of M values and differentially expressed genes were identified with a Benjamini–Hochberg adjusted P value less than 0.05. **b**. Results for individual mice.

Supplementary Data 2. Differentially expressed genes from bulk RNAseq comparing *Ikbkb*$^{mut/mut}$ and *Ikbkb*$^{WT/WT}$ tails. Gene expression was normalized using trimmed mean of M values and differentially expressed genes were identified with a Benjamini–Hochberg adjusted *p*-value < 0.05

Supplementary Data 3. scRNAseq experiment metadata

Supplementary Data 4. Relative representation of Treg subsets by scRNAseq

Supplementary Data 5. Top-ranked differentially expressed genes between *Ikbkb*$^{mut/mut}$ and *Ikbkb*$^{WT/WT}$ from scRNASeq experiment. DEGs were calculated using Seurat FindAllMarkers two-sided Wilcoxon test using Bonferroni correction (adjusted *p*-value cut-off= 0.05).

Supplementary Data 6. Hallmark pathway analysis for NLT skin Treg subset. *p*-values for a collection of gene sets was determined by the fast pre-ranked gene set enrichment analysis (adjusted *p*-value cut-off = 0.05).
