# Supplementary material for: IKK2 controls the inflammatory potential of tissue-resident regulatory T cells in a murine gain of function model
Source: Nat Commun. 2024 Mar 25;15:2345. doi: 10.1038/s41467-024-45870-3 (PMC10963799; doi:10.1038/s41467-024-45870-3)
Supplement: Supplementary file 10 — Reporting Summary [file 41467_2024_45870_MOESM10_ESM.pdf]

nature portfolio

Corresponding author(s): Matthew Cook

Last updated by author(s): 12/12/2023

Reporting Summary

Nature Portfolio wishes to improve the reproducibility of the work that we publish. This form provides structure for consistency and transparency in reporting. For further information on Nature Portfolio policies, see our Editorial Policies and the Editorial Policy Checklist.

Statistics

For all statistical analyses, confirm that the following items are present in the figure legend, table legend, main text, or Methods section.

n/a	Confirmed	
☐	☒	The exact sample size (*n*) for each experimental group/condition, given as a discrete number and unit of measurement
☐	☒	A statement on whether measurements were taken from distinct samples or whether the same sample was measured repeatedly
☐	☒	The statistical test(s) used AND whether they are one- or two-sided<br>*Only common tests should be described solely by name; describe more complex techniques in the Methods section.*
☒	☐	A description of all covariates tested
☐	☒	A description of any assumptions or corrections, such as tests of normality and adjustment for multiple comparisons
☐	☒	A full description of the statistical parameters including central tendency (e.g. means) or other basic estimates (e.g. regression coefficient) AND variation (e.g. standard deviation) or associated estimates of uncertainty (e.g. confidence intervals)
☐	☒	For null hypothesis testing, the test statistic (e.g. *F*, *t*, *r*) with confidence intervals, effect sizes, degrees of freedom and *P* value noted<br>*Give P values as exact values whenever suitable.*
☒	☐	For Bayesian analysis, information on the choice of priors and Markov chain Monte Carlo settings
☒	☐	For hierarchical and complex designs, identification of the appropriate level for tests and full reporting of outcomes
☒	☐	Estimates of effect sizes (e.g. Cohen's *d*, Pearson's *r*), indicating how they were calculated

Our web collection on statistics for biologists contains articles on many of the points above.

Software and code

Policy information about availability of computer code

Data collection	Flow cytometry was performed by BD LSR II or LSR Fortessa and analyzed by Flowjo (v10 Treestar).<br>Blood samples for haematology analysis were performed by the Advia 2120 Haematology Analyser.<br>Serum cytokines were measured by MESO SCALE Discovery Sector Imager 6000 according to the manufacturer's instructions.
Data analysis	Flowjo (v10 Treestar); CellRanger (v6); Seurat (v4); HISAT2 (v2.1.0); FeatureCount (v1.4); Limma (3.54.1); FGSEA (V.1.23.4). Prism (8.0.1). The 10X single cell RNA sequencing data was aligned and quantified using the CellRanger (v6), and then analyzed using Seurat (v4).<br>Bulk RNA sequencing data was assembly by HISAT2 (v2.1.0) and counted based by FeatureCount (v1.4). Gene read libraries were normalized by trimmed mean of M values (TMM) method and transformed by voom, then fitted in a linear model with moderated t-statistics with the empirical Bayes method, and the different expression genes were identified with Benjamini-Hochberg adjusted P value < 0.05, and all statistical analyses were performed by the limma (3.54.1).

For manuscripts utilizing custom algorithms or software that are central to the research but not yet described in published literature, software must be made available to editors and reviewers. We strongly encourage code deposition in a community repository (e.g. GitHub). See the Nature Portfolio guidelines for submitting code & software for further information.

Data

Policy information about availability of data

All manuscripts must include a data availability statement. This statement should provide the following information, where applicable:

- Accession codes, unique identifiers, or web links for publicly available datasets
- A description of any restrictions on data availability
- For clinical datasets or third party data, please ensure that the statement adheres to our policy

Single cell RNA-sequencing data and bulk RNA-sequencing data were deposited to the NCBI and accessible under the GEO accession number GSE228385 and GSE248917, respectively.
Data are available as of the date of publication. Further information and requests for resources and reagents should be directed to the lead contact, Matthew Cook (mc2386@cam.ac.uk).

Research involving human participants, their data, or biological material

Policy information about studies with human participants or human data. See also policy information about sex, gender (identity/presentation), and sexual orientation and race, ethnicity and racism.

Reporting on sex and gender	n/a
Reporting on race, ethnicity, or other socially relevant groupings	n/a
Population characteristics	n/a
Recruitment	n/a
Ethics oversight	n/a

Note that full information on the approval of the study protocol must also be provided in the manuscript.

Field-specific reporting

Please select the one below that is the best fit for your research. If you are not sure, read the appropriate sections before making your selection.

- [x] Life sciences
- [ ] Behavioural & social sciences
- [ ] Ecological, evolutionary & environmental sciences

For a reference copy of the document with all sections, see nature.com/documents/nr-reporting-summary-flat.pdf

Life sciences study design

All studies must disclose on these points even when the disclosure is negative.

Sample size	No statistical analysis was used to predetermine the sample size. Sample sizes were chosen based on availability and fundation.
Data exclusions	No sample was excluded from the analyses.<br>In single cell RNA sequencing analysis, after clustering the integrated dataset (Treg), we excluded a tiny B cell population characterised by high expression of B cell-related genes (e.g. Cd19, Cd79a). An additional population that lacked expression of T cell-related genes (e.g. Cd3e, Cd4) was also excluded.
Replication	All experiments were repeated at least 2 or 3 times as described in figure legends. All replication attempts were repeatable and reproducible with consistent observations. ScRNAseq and bulk RNAseq was performed with three biological replicates.
Randomization	Nil. Mice were used according to genotype and matched for age and sex. In most cases, littermate controls were used
Blinding	Histology, cytokine analysis, and flow cytometry acquisition was performed with blinding to genotype. Experimental method prevented blinding for scRNAseq and bulk RNAseq analysis

Reporting for specific materials, systems and methods

We require information from authors about some types of materials, experimental systems and methods used in many studies. Here, indicate whether each material, system or method listed is relevant to your study. If you are not sure if a list item applies to your research, read the appropriate section before selecting a response.

Materials & experimental systems

n/a	Involved in the study
☐	☒ Antibodies
☒	☐ Eukaryotic cell lines
☒	☐ Palaeontology and archaeology
☐	☒ Animals and other organisms
☒	☐ Clinical data
☒	☐ Dual use research of concern
☒	☐ Plants

Methods

n/a	Involved in the study
☒	☐ ChIP-seq
☐	☒ Flow cytometry
☒	☐ MRI-based neuroimaging

Antibodies

Antibodies used

The following antibodies were used: 7AAD (Thermo Fisher, cat no. A1310, dilution 1:1000), Rat anti-CD16/CD32 monoclonal antibody unconjugated (BD Biosciences, cat no. 553142, RRID: AB_394657, clone 2.4G2, dilution 1:50), Brilliant violet 510 anti-mouse CD3 (BioLegend, cat no. 100234, RRID: AB_2562555, clone 17A2, dilution 1:100), Alexa fluor 700 anti-mouse CD19 (BioLegend, cat no. 115528, RRID: AB_493735, clone 6D5, dilution 1:200), Brilliant violet 605 anti-mouse CD45.1 (BioLegend, cat no. 110737, RRID: AB_11204076, clone A20, dilution 1:100), Brilliant violet 510 anti-mouse CD45.2 (BioLegend, cat no. 109838, RRID: AB_2650900, clone 104, dilution 1:100), APC-Cy7 anti-mouse CD45 (BioLegend, cat no. 103116, RRID: AB_312981, clone 30-F11, dilution 1:200), FITC anti-mouse CD4 (BioLegend, cat no. 100510, RRID: AB_312713, clone RM4-4, dilution 1:800), PE anti-mouse CD4 (Thermo Fisher, cat no. 12-0042-83, RRID: AB_465511, clone RM4-4, dilution 1:400), Alexa fluor 700 anti-mouse CD8a (BioLegend, cat no. 100729, RRID: AB_493702, clone 53-6.7, dilution 1:400), Brilliant violet 605 anti-mouse CD25 (BioLegend, cat no. 102035, RRID: AB_2563059, clone PC61, dilution 1:100), PE-Cy7 anti-mouse/human CD44 (BioLegend, cat no. 103029, RRID: AB_830786, clone IM7, dilution 1:500), APC/Fire 750 anti-mouse CD62L (BioLegend, cat no. 104449, RRID: AB_2629772, clone MEL-14, dilution 1:800), APC anti-mouse CD103 (Thermo Fisher, cat no. 17-1031-82, RRID: AB_1106992, clone 2E7, dilution 1:100), Alexa fluor 647 anti-mouse FOXP3 (BioLegend, cat no. 126407, RRID: AB_1089115, clone MF-14, dilution 1:100), PE/Dazzle 594 anti-mouse/human Helios (BioLegend, cat no. 137231, RRID: 2565797, clone 2276, dilution 1:100), APC conjugated Rat anti-IFN-gamma (BD Biosciences, cat no. 554413, RRID: AB_398551, clone XMG1.2, dilution 1:800), FITC anti-mouse IL-17A (BioLegend, cat no. 506908, RRID: AB_536009, clone TC11-18H10.1, dilution 1:200), Brilliant violet 510 anti-mouse Ly6G (BioLegend, cat no. 127633, RRID: AB_2562937, clone 1A8, dilution 1:50), TCR gamma/delta eFluor 450 (Invitrogen, cat no. 48-5711-82, RRID: AB_2574071, clone eBioGL3, dilution 1:50), BV421 mouse anti-Ki-67 (BD Biosciences, cat no. 562899, RRID: AB_2686897, clone B56, dilution 1:100), Alexa fluor 700 anti-mouse CD45.1 (BioLegend, cat no. 110724, RRID: AB_493733, clone A20, 1:200), BUV737 Ms CD45R/B220 (BD Biosciences, cat no. 612838, RRID: AB_2870160, clone 104, dilution 1:100), Rat anti-CD19 monoclonal antibody PE (BD Biosciences, cat no. 553786, RRID: AB_395050, clone 1D3, dilution 1:200), PerCP/Cyanine5.5 anti-mouse CD4 (BioLegend, cat no. 100433, RRID: 893330, clone GK1.5, dilution 1:500), BUV395 Rat anti-mouse CD8a (BD Biosciences, cat no. 563786, RRID: AB_2732919, clone 53-6.7, dilution 1:200), Rat anti-CD25 APC monoclonal antibody (BD Biosciences, cat no. 557192, RRID: 398623, clone PC61, dilution 1:200), FOXP3 monoclonal antibody FITC (Thermo Fisher, cat no. 11-5773-82, RRID: AB_465243, clone FJK-16S, dilution 1:200), PerCP-Cyanine5.5 anti-mouse CD3 (BioLegend, cat no. 100218, RRID: AB_1595492, clone 17A2, dilution 1:100), Brilliant violet 605 anti-mouse CD4 (BD Biosciences, cat no. 563151, RRID: AB_2687549, clone RM4-5, dilution 1:200), Pacific blue anti-mouse/human CD44 (BioLegend, cat no. 103020, RRID: AB_493683, clone IM7, dilution 1:200), Brilliant violet 711 anti-mouse CD25 (BioLegend, cat no. 102049, RRID: AB_2564130, clone PC61, dilution 1:400), PE anti-mouse CD103 (BioLegend, cat no. 121405, RRID: AB_535948, clone 20000000, dilution 1:100), IL-17A monoclonal antibody APC (Thermo Fisher, cat no. 17-7177-81, RRID: AB_763580, clone eBio17B7, dilution 1:200), IFN gamma monoclonal antibody PE-Cyanine7 (Thermo Fisher, cat no. 25-7311-82, RRID: AB_469680, clone XMG1.2, dilution 1:1000), Rat anti-CD4 monoclonal antibody Alexa fluor 700 (BD Biosciences, cat no. 557956, RRID: AB_396956, clone RM4-5, dilution 1:800), Ms CD45.1 BUV737 (BD Biosciences, cat no. 612811, RRID: AB_2870136, clone A20, dilution 1:100), Rat anti-CD19 APC (BD Biosciences, cat no. 550992, RRID: AB_398483, clone 1D3, dilution 1:400), Brilliant violet 605 anti-mouse CD62L (BioLegend, cat no. 104438, RRID: AB_2563058, clone MEL-14, dilution 1:800).

Validation

All antibodies were from commercial sources and validated by manufacturers. Research Resource Identifiers (#RRID) confirming validation and linking to clone, clonality and species from manufacturers' websites are provided above.

Animals and other research organisms

Policy information about studies involving animals; ARRIVE guidelines recommended for reporting animal research, and Sex and Gender in Research

Laboratory animals

Wild-type and IkbkbV203I mice with a C57BL/6 background of 6 weeks to 12 months of age were used. When possible, mice were age-matched within experiments.

Wild animals

No wild animals were used.

Reporting on sex

A combination of male and female mice were used for experiments. No distinct differences between sexes were observed.

Field-collected samples

The study did not involve any field-collected samples.

Ethics oversight

Animals were used in agreement with the protocols approved by the ANU Animal Experimentation Ethics Committee. This research operated under ANU Ethics Protocols A2018/06, A2020/21, and A2021/22.

Note that full information on the approval of the study protocol must also be provided in the manuscript.

Plants

Seed stocks	n/a
Novel plant genotypes	n/a
Authentication	n/a

Flow Cytometry

Plots

Confirm that:

- [x] The axis labels state the marker and fluorochrome used (e.g. CD4-FITC).
- [x] The axis scales are clearly visible. Include numbers along axes only for bottom left plot of group (a 'group' is an analysis of identical markers).
- [x] All plots are contour plots with outliers or pseudocolor plots.
- [x] A numerical value for number of cells or percentage (with statistics) is provided.

Methodology

Sample preparation	Mouse lymphoid organs (spleen and lymph nodes) were collected in cold RPMI-640 (ThermoFisher) with 10% FBS (ThermoFisher). Spleen and lymph nodes were disrupted and processed into single cell suspension by syringe plunger and passaged through 70um filters (Miltenyi Biotec), followed by red blood cell lysis process (BD).<br><br>For bone marrow harvest, the tibia and femur were cleaned and cut at both ends to allow for bone marrow to be flushed out. These bones were collected into empty 200L microcentrifuge tubes; these tubes had a small hole at the bottom tip which was created by pushing a 16-gauge needle through. These 200L tubes were then placed into 1.5mL microcentrifuge tubes with lid removed. Samples were centrifuged at high speed for 10 seconds which enabled bone marrow to be flushed out to the bottom of the 1.5mL tubes. Cell pellets were then resuspended in culture media and passed through a 70um cell strainer to prevent cell clumping.
Instrument	BD LSR II or LSR Fortessa
Software	BD FACSDiva. Flowjo (v10 Treestar).
Cell population abundance	Cells sorting was achieved with > 95% single cell population purity.
Gating strategy	Cells were gated by lymphocytes by FSC, SSC, doublets were excluded by FSC-H/FSC-A and SSC-W/SSC-H, live cells were gated by FSC/viability, gating strategy for different cells was provided in main text and method sections, such as Treg (CD4+CD8-FOXP3-GFP+) cell population for single cell RNA sequencing, naive T cells (CD4+CD62LhiCD44lo CD25-), APCs (CD3-CD4-CD8-) and Tregs (CD4+CD25+Foxp3-GFP+) for in vitro Treg suppression assay.

- [x] Tick this box to confirm that a figure exemplifying the gating strategy is provided in the Supplementary Information.
